# Purification and Characterization of Polyphenol Oxidase in the Fruits of *Opuntia ficus-indica*

**DOI:** 10.3390/biology12101339

**Published:** 2023-10-16

**Authors:** Dudu Demir, Selda Kabak, Kardelen Çağlayan

**Affiliations:** Department of Agricultural Biotechnology, Faculty of Agriculture, Isparta University of Applied Sciences, 32260 Isparta, Türkiye; yl2230639001@isparta.edu.tr (S.K.); kardelencaglayan@isparta.edu.tr (K.Ç.)

**Keywords:** affinity chromatography, characterization, polyphenol oxidase, prickly pear, purification

## Abstract

**Simple Summary:**

*Opuntia ficus-indica* (L.) Mill. (OFI) is a member of the Cactaceae family. Its fruits are available in a wide range of colors, including orange, lime green, purple, and red. In many countries, the cacti are cultivated and used as foods and medicinal plants. They can grow in dry and semi-arid environments and have substantial economic potential. In the current study, for the first time, polyphenol oxidase (PPO) was purified from the fruits of *O. ficus-indica*, whose color was orange, through utilizing the Sepharose 4B–L-tyrosine–*p*-aminobenzoic acid column, and the purified enzyme was characterized.

**Abstract:**

Firstly, polyphenol oxidase (PPO) was purified from the fruits of *Opuntia ficus-indica* using Sepharose 4B–L-tyrosine–*p*-aminobenzoic acid affinity chromatography, and the enzyme was characterized. The PPO was purified 20.59-fold. Thereafter, PPO was performed on sodium dodecyl sulfate–polyacrylamide gel electrophoresis (SDS-PAGE). The kinetic parameters, optimum pHs, and optimum temperatures were investigated for three substrates. *Opuntia ficus-indica* PPO’s optimum pH and optimum temperature were 9.0 and 20 °C; 7.5 and 20 °C; and 7.5 and 30 °C, respectively, when using pyrogallol, catechol, and 4-methyl catechol as substrates. For the pyrogallol, catechol, and 4-methyl catechol, the *Km*, *Vmax*, and *Vmax*/*Km* values were determined as 16.67 mM, 833.33 U/mLmin, and 50 U/mLminmM; 6.33 mM, 126.58 U/mLmin, and 20 U/mLminmM; and 5.38 mM, 107.53 U/mLmin, and 20 U/mLminmM, respectively. As a result, pyrogallol was a more appropriate substrate than catechol and 4-methyl catechol for the PPO from *Opuntia ficus-indica*.

## 1. Introduction

*Opuntia ficus-indica* (L.) Mill. is a dicotyledonous angiosperm plant that is known as the nopal cactus or prickly pear. It is a Cactaceae family member, which has approximately 130 genera and 1500 species. This plant has significant economic potential and can thrive in dry and semi-arid environments all over the world [1]. There are many places in the world where the soil and climate are ideal for the growth of cacti. The true jewels of arid and semiarid climates are cactus fruits [2]. This cactus (OFI) originated in Mexico and spread to Central America and the southern United States. Eventually, it also appeared in Africa, Asia, and southern Europe. In more than 30 countries, this cactus is cultivated and used as a food and medicinal plant; in South Africa and Australia, it is considered a poisonous weed. Cactus cultivation occupies large areas in Chile, Algeria, Mexico, and Brazil. Cacti are considered an alternative or substitute food during periods of drought, as they remain juicy and fresh for a long time [3]. As a result of the presence of some bioactive substituents, the pulp’s sweet and juicy texture offers intriguing health-promoting properties [2]. *Opuntia ficus-indica* is mostly utilized as a food plant in the Mediterranean basin due to its edible fruit and as a supplement to animal feed [1]. There are numerous uses for plants in the genus *Opuntia*. Carminic acid production is followed by food use, functional qualities, and other uses. The fruits, sometimes known as prickly pears and nopal, are two plant parts that have been eaten as food. The prickly pears can be eaten raw, dried in the sun, or made into marmalades. Mexican regions utilize nopal as an ingredient in salads [4]. For the treatment of diarrhea, rheumatism, ulcers, asthma, gonorrhea, wounds, indigestion, and burns, every vegetative part of *Opuntia* sp. has traditionally been utilized as a valuable nutrient and in folkloric medicines. In modern nutrition, medicine, and pharmacology, they are seldom used, though [5]. Several parts of *O. ficus-indica* species have been found to have diuretic and antigonococcal properties, according to scientific studies [4,6]. *Opuntia ficus-indica* species fruits have analgesic and anti-inflammatory properties [4,7]. Research has shown that the nopal of *O. ficus-indica* has anti-ulcer properties [4,8]. Additionally, the fruits of *Opuntia dillenii* and the nopal of *O. ficus-indica* have been shown to have hypocholesterolemic and antihyperglycemic properties [4,9,10]. *Opuntia* is a significant source of bioactive pigments, such as betalines, betateinines, and anthocyanins [11]. In semi-arid parts of California, South America, the Mediterranean, and Mexico, cultivated cultivars of cactus pears (*Opuntia ficus-indica* L. Mill.) and associated species produce 120–180 g fruits with total soluble solid values of 12–16%. The fruits are available in a wide range of colors, including orange, lime green, purple, and red [12].

Polyphenol oxidase enzymes are preferred in nutritional, environmental, biomedical, and pharmaceutical applications, for example, personal care, paper, cosmetics, food, textiles, wastewater treatment, analyses of biological fluids, the production of biofuel cells, and biosensors using the detection of pesticide residues [13]. PPO enzymes, which are present in large quantities in all vegetables and fruits, are one of the most crucial enzymes in plants [14]. According to their distinct substrates and mechanisms of action, PPOs can be divided into three primary categories: catecholase (EC 1.10.3.1), laccase (EC 1.10.3.2), and tyrosinase (EC 1.14.18.1) [15]. The copper-containing oxidoreductase enzyme polyphenol oxidase catalyzes *o*-hydroxylation of monophenols to *o*-diphenols and also exhibits additional catalytic oxidation of *o*-dihydroxy phenols to *o*-quinones [14]. Polyphenol oxidase is also responsible for browning in plants and animal melanization [16]. Enzymatic browning is a known and significant problem due to the decreasing shelf-life, flavor, appearance, and nutrient value of many fruits, including *Opuntia ficus-indica*. Thus, it is substantial to characterize the PPO enzyme that causes browning. Polyphenol oxidase enzymes have been extracted, purified, and characterized in various fruits and vegetables, such as mulberry (*Morus alba* L.) [17], Cimin Grape (*Vitis vinifera* spp., Cimin) [18], royal date fruit [19], *Musa acuminata* fruit pulp [20], myrtle berries (*Myrtus communis* L.) [21], Damson plum [13], and dried *Volvariella bombycina* [22]. Although studies about the PPO enzymes of many fruits have been found, the purification and characterization of *O. ficus-indica* fruits’ PPO are not present in the literature. With our research, the PPO from OFI was studied for the first time.

The objective of this research was to extract, purify, and characterize the PPO enzyme from *O. ficus-indica* fruits. Following the PPO enzyme’s purification by utilizing Sepharose 4B–L-tyrosine–*p*-aminobenzoic acid column chromatography, its biochemical properties, such as its kinetic characteristics (*Km* and *Vmax*), substrate specificity, optimum pH, and temperature, were established. The PPO enzyme of *O. ficus-indica* was subjected to SDS-PAGE.

## 2. Materials and Methods

### 2.1. Collection of Plant Material

Fruits of *O. ficus-indica*, whose color was orange, were obtained from a field in the Kepez district of Antalya province in Türkiye. The fruit was harvested, immediately transported to the laboratory (avoiding all physical damage) and used fresh for PPO.

### 2.2. Chemicals

Sodium chloride (NaCl), di-potassium hydrogen phosphate (K_2_HPO_4_), polyethylene glycol 4000 (PEG), tris(hydroxymethyl)aminomethane (TRIS), hydrochloric acid 37% (HCl), sodium hydroxide (NaOH), ammonium sulfate ((NH_4_)_2_SO_4_), glycerol, di-sodium hydrogen phosphate (Na_2_HPO_4_), bovine serum albumin (BSA), β-mercaptoethanol, acetic acid (CH_3_CO_2_H), catechol, ortho-phosphoric acid 85% (H_3_PO_4_), ethanol, sodium nitrite (NaNO_2_), and Coomassie Brilliant Blue G 250 were purchased from Merck (Darmstadt, Germany). Bromophenol blue (3′, 3″, 5′, 5″-Tetrabromo phenolsulfonphthalein) was purchased from Fisher Scientific (Loughborough, UK). The protein molecular weight marker was purchased from Thermo Scientific (Vilnius, Lithuania). Coomassie Brilliant Blue R-250 was purchased from Amresco Inc. (Solon, OH, USA). Sepharose 4B, L-tyrosine, *p*-aminobenzoic acid, L-ascorbic acid, cyanogen bromide solution (CNBr), glycine, ammonium persulfate, sodium bicarbonate (NaHCO_3_), dialysis sack (dialysis tubing cellulose membrane avg. flat width 25 mm (1.0 in.)), sodium dodecyl sulfate (SDS), 30% solution of acrylamide/bis-acrylamide, *N*,*N*,*N*′,*N*′-tetramethyl ethylenediamine (TEMED), pyrogallol, and 4-methyl catechol were purchased from Sigma-Aldrich Co. Ltd. (Saint Louis, MO, USA). To purify the PPO enzyme in this research, Sepharose 4B–L-tyrosine–*p*-aminobenzoic acid gel was synthesized in the Enzyme and Microbial Biotechnology Laboratory at Isparta University of Applied Sciences (Isparta, Türkiye) according to the method by Arslan et al. [17].

### 2.3. Extraction and Purification of the Enzyme

*O. ficus-indica* fruits’ PPO extraction and purification were performed using a method modified from Arslan et al. [17]. The polyphenol oxidase enzyme was purified from cactus by utilizing a Sepharose 4B–L-tyrosine–*p*-aminobenzoic acid column. Firstly, the fruit samples were washed with sufficient sterile distilled water, the air-dried cactus fruits were quickly peeled thinly, and the study continued with the flesh of the cactus fruits for preparing the crude extract. The flesh of cactus fruits (50 g) was separated quickly into thin slices and homogenized by a stick blender for 2 min with 100 mL of a 0.5 M (pH 7.3) phosphate buffer solution, including 10 mM ascorbic acid and 0.5% polyethylene glycol. The homogenate was filtered through a cheesecloth to remove the core and plant cell walls and the cellulosic fibrous part, and then the filtrate was centrifuged for 30 min at 4 °C at 15,000 rpm. After the centrifugation process, the precipitate containing the plant cell walls and the cellulosic fibrous part remaining from the filtration process was discarded, and the supernatant part obtained was used as the crude extract. The supernatant was precipitated with solid ammonium sulfate at 80% saturation, and the precipitate was collected through centrifugation for 40 min at 15,000 rpm at 4 °C, redissolved in a 5 mM (pH 6.30) phosphate buffer solution, and dialyzed against the same buffer solution. This solution was applied to the affinity column, which was pre-equilibrated with a 50 mM (pH 5.0) phosphate buffer solution. This buffer solution was utilized to wash out the gel. The PPO enzyme was eluted with a 50 mM (pH 7.0) phosphate buffer solution/1 M NaCl.

### 2.4. PPO Activity Test

Using a UV-Visible spectrophotometer (Spectramax-Plus 384, Molecular Devices, LLC., San Jose, CA, USA), the PPO activity of *O. ficus-indica* was determined by measuring the absorbance increase for one minute according to the modified method of Arslan et al. [17]. Pyrogallol, catechol, and 4-methyl catechol were used as test substrates. The sample cuvette contained 0.83 mL of 0.1 M phosphate buffer solution, 0.13 mL of 0.1 M substrate solution, and 0.04 mL of the enzyme solution. The blank solution included only the substrate solution (1.0 mL). The measurement was performed to an absorbance of 320 nm for pyrogallol [23] and 420 nm for 4-methyl catechol and catechol [17] by using a spectrophotometer. In triplicate measurements, the PPO activity was assayed. One unit of PPO activity was defined as the amount of enzyme that caused a change in absorbance of 0.001 mL^−1^min^−1^.

### 2.5. Detection of Protein Concentration

The protein concentration was detected using the Bradford method at 595 nm. Bovine serum albumin was used as the standard [24]. In affinity chromatography, the protein was determined by absorbance at 280 nm [17].

### 2.6. Electrophoresis of PPO

Sodium dodecyl sulfate-polyacrylamide gel electrophoresis of the purified PPO from *O. ficus-indica* was applied according to the Laemmli method [25]. The purified PPO of *O. ficus-indica* was subjected to SDS-PAGE in a Mini Protean Tetra Cell Electrophoresis Unit (Bio-Rad Laboratories, Hercules, CA, USA), with 3% stacking gel and 10% running gel. The purified PPO by affinity gel from *O. ficus-indica* and standard proteins were run on the SDS-PAGE gel. The purified PPO and marker were loaded in lanes, and the slab gels were 1 mm thick. The PPO and marker were run at 80 V in the stacking gel and 150 V in the separating gel (running gel). After the electrophoresis, the protein bands were visualized using Coomassie Brilliant Blue R-250. The protein molecular weight marker (Thermo Scientific, Lithuania), which was used for comparison to the molecular weight, included lysozyme (14.4 kDa), β-lactoglobulin (18.4 kDa), REase Bsp98l (25.0 kDa), lactate dehydrogenase (35.0 kDa), ovalbumin (45.0 kDa), bovine serum albumin (66.2 kDa), and β-galactosidase (116.0 kDa).

### 2.7. Effects of pH on the PPO Activity

To determine the optimum pH for the PPO activity of *O. ficus-indica*, the PPO activities were measured at different pH values: 0.1 M Tris-base buffers (pH 8.5–9.0), 0.1 M phosphate buffers (pH 6.0–8.0), and 0.1 M sodium acetate buffers (pH 4.5–5.5) at different temperatures in the range of 15–45 °C using three different substrates (0.1 M of pyrogallol, 0.1 M of 4-methyl catechol, and 0.1 M of catechol). Each measurement was repeated in triplicate, and the values were averaged.

### 2.8. Effects of Temperature on the PPO Activity

The optimum temperature for the PPO activity of *O. ficus-indica* was detected to range from 15 to 45 °C utilizing three different substrates (0.1 M of pyrogallol, 0.1 M of catechol, and 0.1 M of 4-methyl catechol) and different pH values in the range of 4.5–9.0. Each measurement was repeated in triplicate, and the values were averaged.

### 2.9. Enzyme Kinetics Studies

The maximum velocity (*Vmax*) and Michaelis–Menten constant (*Km*) values for the PPO of *O. ficus-indica* were detected by measuring the enzyme activity under optimum temperature and pH conditions at different concentrations of substrates for pyrogallol, 4-methyl catechol, and catechol through Lineweaver-Burk plots [26]. Each measurement was repeated in triplicate, and the values were averaged.

## 3. Results and Discussion

### 3.1. Extraction and Purification of the Enzyme

Although PPO has been purified and characterized from many species’ fruits, there is no report on PPO from *O. ficus-indica* fruit. After the research on this topic was examined, Sepharose 4B–L-tyrosine–*p*-aminobenzoic acid chromatography was utilized for the first time to purify PPO from *O. ficus-indica* fruit. The polyphenol oxidase purification steps are provided in Table 1. The most commonly used chromatographic technique is affinity chromatography because it is practical. The degree of purification offered by chromatographic techniques is also very high. A more efficient technique is affinity chromatography based on immobilized (through 4-aminobenzoic acid) tyrosine–Sepharose [22]. The specific activity of the PPO enzyme found 1575.72 U/mg protein in the crude extract, whereas it had 32,449.13 U/mg protein in the affinity purification. These findings show a 20.59-fold purification of the enzyme. Using the same chromatographic method previously, the PPO enzyme was purified 74-fold from mulberry (*Morus alba* L.) [17], 11.2-fold from Cimin grape (*Vitis vinifera* spp., Cimin) [18], 93.88-fold from Damson plum [13], and 33.85-fold from dried *Volvariella bombycina* [22]. Using the same chromatographic technique, different purification levels from several plant species were achieved.

Furthermore, PPO can be purified from a variety of sources using a variety of chromatography methods. PPO was purified 8-fold by Sepharcyl S-200 from royal date fruit [19], 4.04-fold by gel filtration chromatography from *Musa acuminata* fruit pulp [20], 10.46-fold through Sepharose 6B–L-tyrosine–*p*-aminobenzoic acid affinity gel from Damson plum [13], and 5.5-fold by DEAE–Sephacel chromatography from myrtle berries (*Myrtus communis* L.) [21].

**Figure 1:**
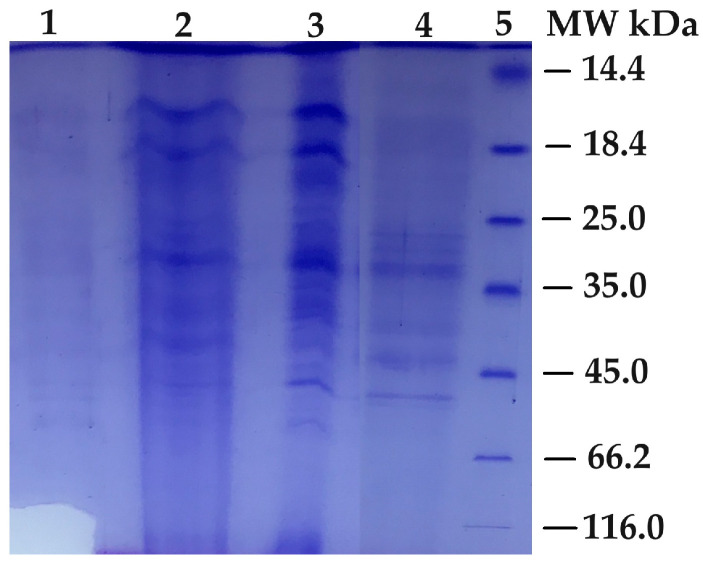
Analysis of the *O. ficus-indica* PPO SDS-PAGE: (**1**) crude enzyme extract; (**2**) after ammonium sulfate; (**3**) after dialysis; (**4**) after affinity purification; (**5**) molecular marker.

The PPO from *O. ficus-indica* that was eluted in the chromatographic separations was assessed by SDS-PAGE. Figure 1 displays the results of the Coomassie Brilliant Blue R-250 protein staining. According to that, many bands were observed in each lane. The appearance of many bands may have arisen from the presence of polyphenol oxidase and other enzymes (lanes 1, 2, and 3). The bands in lane 1 in the SDS-PAGE appeared as thin strips due to the low concentration of crude extract. Generally, the amount of protein was minimal, which describes the appearance of thin strips. The amount of total protein (3.7126 mg) was abundant in the crude enzyme step due to containing many protein types and a high volume (130 mL). Further, the amount of total protein was 1.1007 mg in the ammonium sulfate precipitation step. However, the concentration of the protein in the crude enzyme step was lower than in the ammonium sulfate precipitation step. Because the protein was precipitated in the ammonium sulfate precipitation step and redissolved in the minimum volume of buffer (5 mL), it could dissolve. The lower volume increased the protein concentration. 

During the purification steps, the types of proteins gradually decreased. Hence, the number of bands was gradually reduced. Figure 1 shows that the number of bands in lane 4 was the lowest. A few bands were observed, of which two bands slightly smaller than 35 kDa and slightly larger than 45 kDa were much more prominent in lane 4. According to SDS-PAGE studies on other plants, the molecular weights of three isoenzymes of royal date PPO were 20 kDa, 45 kDa, and 64 kDa [19]. The three novel active PPO isoforms of banana were detected to be 28 kDa, 45 kDa, and 65 kDa by gel filtration chromatography and SDS-PAGE [20]. The molecular weight of PPO from the Kirmizi Kismis grape was found to be approximately 38.1 kDa by SDS-PAGE as a single band [27]. Looking at the results in the literature, it is seen that there are bands with different molecular weights depending on the source of the PPO enzyme.

### 3.2. Effects of Temperature and pH on the PPO Activity

Conditions like temperature and pH have a significant impact on the activity of enzymes. The activity of *O. ficus-indica* PPO was measured at different temperatures and pHs using pyrogallol, 4-methyl catechol, and catechol as test substrates. pH is a determinant factor for the statement of enzymatic activity. The pH profile of PPO was measured in this study at 4.5–9.0 with 0.5 pH unit intervals. Another significant element that affects the effectiveness of enzyme catalysis is temperature. Due to thermal denaturation, which occurs as the temperature rises, the reaction rate often decreases at high temperatures. The effect of temperature on *O. ficus-indica* PPO activity was studied in the range of 15–45 °C with 5 °C unit intervals. Pyrogallol, catechol, and 4-methyl catechol substrates were utilized to detect the *O. ficus-indica* PPO’s optimum pH and temperature. Table 2 displays *O. ficus-indica* PPO’s enzymatic profile at various pH levels and temperatures. The optimum pH and temperature values of the PPO enzyme of *O. ficus-indica* were obtained to be 9.0 and 20 °C; 7.5 and 30 °C; and 7.5 and 20 °C, respectively, by utilizing pyrogallol, 4-methyl catechol, and catechol as substrates, as summarized in Table 2. According to the results, PPO activity tended to decrease and then increase, and the optimal pH changed rapidly even with a temperature difference of 5 degrees. The variability in the pH and temperature could be explained as effects of the existence of PPO isoenzymes. The differential enzymatic properties of these isoforms propose that they might have various physiological functions [19]. The findings in this study have similarities with the results obtained by [17,19,21,23,28,29].

The values changed according to the substrate used. The enzyme activity and changes exhibited a significant dependency on the pH and temperature values and the type of substrate [19,21]. Substrates with different structures were used in this study. For instance, the structures of two o-diphenolic substrates utilized (4-methyl catechol and catechol) differ from the structure of pyrogallol as a polyphenolic substrate (1,2,3-trihydroxybenzene). The values of the optimum pH and temperature are relative to the binding, complementarity, and catalysis degree of the enzyme towards its substrate in the transition state [19]. The optimum pH and temperature were found to be 6.8 and 30 °C for myrtle berries PPO [21], 6.5 and 40 °C for *Musa acuminata* fruit pulp PPO [20], and 7.2 and 25 °C for Damson plum PPO [13], in the presence of catechol as a substrate; 5.0 and 30 °C for Kirmizi Kismis grape PPO [27], and 4.5 and 10 °C for Damson plum PPO [13], in the presence of 4-methyl catechol as a substrate; 7.0 and 25 °C for pomegranate arils (*Punica granatum* L. cv. Wonderful) PPO [30], and 6.8 and 5 °C for Damson plum PPO [13], in the presence of pyrogallol as a substrate. It is reported that royal date PPOs with pyrocatechol (catechol), pyrogallol, and 4-methyl catechol substrates had maximum activity at pH 5.6, 5.6 and 8, and 4–5.6, respectively [19]. As can be seen here, when the results in the literature are searched, it can be seen that the optimum pH and optimum temperature values differ according to the substrate and the PPO enzyme source.

### 3.3. Kinetic Parameters

**Figure 2:**
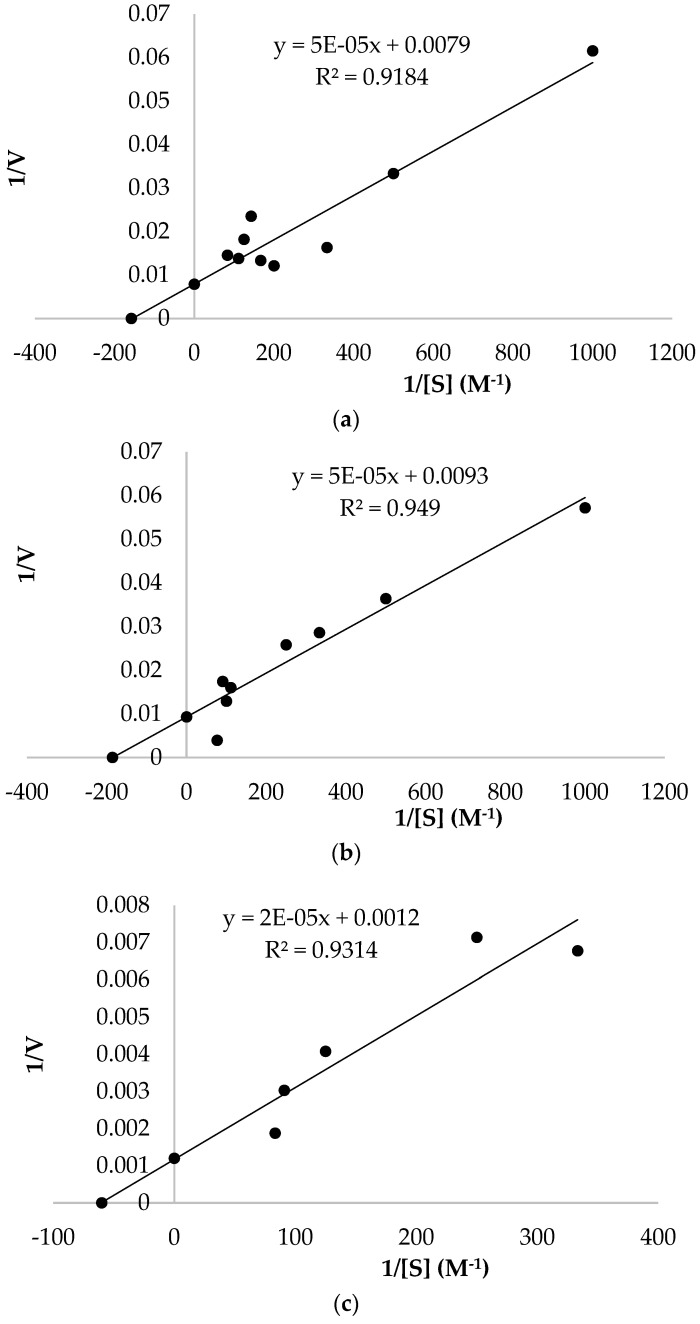
Lineweaver–Burk plots of *O. ficus-indica* (**a**) catechol; (**b**) 4-methyl catechol; (**c**) pyrogallol.

Lineweaver–Burk plots for 4-methyl catechol, pyrogallol, and catechol were utilized to calculate the maximum velocity *Vmax*, Michaelis–Menten constant *Km*, and *Vmax*/*Km* value of PPO in *O. ficus-indica* under optimum temperature and optimum pH conditions (Figure 2). Utilizing catechol, 4-methyl catechol, and pyrogallol substrates, *Km*, *Vmax*, and *Vmax*/*Km* of *O. ficus-indica*’s PPO were detected to be 6.33 mM, 126.58 U/mLmin, and 20 U/mLminmM; 5.38 mM, 107.53 U/mLmin, and 20 U/mLminmM; and 16.67 mM, 833.33 U/mLmin, and 50 U/mLminmM, respectively (Table 3). In the literature, *Km* values for the PPO enzyme have been found to be in the range of 1–10 mM [31]. According to the results, the *Km* values for the catechol and 4-methyl catechol substrates of the PPO enzyme correspond with many studies in the literature. In contrast, the *Km* value for the pyrogallol substrate of the PPO enzyme was found to be higher than the value range. A higher *Km* indicates a lower substrate affinity, and a lower *Km* indicates a higher substrate affinity for the enzyme. The results show that among the three substrates, 4-methyl catechol had the lowest *Km* value towards *O. ficus-indica*’s PPO (5.38 mM), and the enzyme had a higher affinity for 4-methyl catechol. This was followed by the catechol substrate, with a *Km* value of 6.33 mM. Pyrogallol had the highest *Km* value and lowest affinity among the used substrates. The *Vmax* of an enzyme represents its catalytic activity. *Vmax* is directly proportional to its activity. A higher *Vmax* indicates a higher catalytic activity, and a lower *Vmax* indicates a lower catalytic activity for the enzyme. Pyrogallol had the highest *Vmax* value towards *O. ficus-indica*’s PPO (833.33 U/mLmin), followed by catechol (126.58 U/mLmin) and 4-methyl catechol (107.53 U/mLmin). Accordingly, pyrogallol had the highest catalytic activity among the substrates. The substrates appropriate for an enzyme can be assessed with the highest catalytic efficiency (*Vmax*/*Km*). Accordingly, the *Vmax*/*Km* (U/mLminmM) ratio for pyrogallol was 50.00, followed by 20.00 for catechol and 4-methyl catechol. Therefore, pyrogallol appears to be the most suitable substrate for PPO from OFI. The *Vmax* and *Km* values were determined to be 4.1 U/mLmin and 3.34 mM for myrtle PPO [21]; 37.7 mM and 20,534.1 U/mLmin for royal date fruit PPO [19], with catechol as a substrate; 4.8 mM and 2000.0 U/mLmin for Kirmizi Kismis grape PPO [27]; 1.9 mM and 13,414.8 U/mLmin for royal date fruit PPO [19], with 4-methyl catechol as a substrate; 35.3 mM and 3796.7 U/mLmin for royal date fruit PPO [19], with pyrogallol as a substrate. The large range in the PPO’s *Km* and *Vmax* values found in this research and reported in the literature may be due to a number of factors, including the substrate, different extraction pHs, variety, nutrient sources, buffer solution, purity of the enzyme extract, and different assay methods used.

## 4. Conclusions

The objective of this research was to purify and characterize the PPO enzyme in the fruits of *O. ficus-indica*, whose color was orange. The PPO from *O. ficus-indica* was isolated and purified. The purification processes included extraction, ammonium sulfate precipitation, dialysis, and affinity column chromatography. In this study, the optimum temperature and pH were determined for each of the pyrogallol, catechol, and 4-methyl catechol substrates. The optimum pH and temperature for *O. ficus-indica* PPO were found to be 7.5 and 20 °C; 7.5 and 30 °C; and 9.0 and 20 °C, respectively, when utilizing catechol, 4-methyl catechol, and pyrogallol as substrates. In the research, Lineweaver–Burk plots were plotted, showing the activity of *O. ficus-indica* PPO as a function of various concentrations of catechol, 4-methyl catechol, and pyrogallol as substrates and under optimum conditions (pH and temperature). For catechol, 4-methyl catechol, and pyrogallol, the *Km* and *Vmax* values were determined to be 6.33 mM and 126.58 U/mLmin; 5.38 mM and 107.53 U/mLmin; and 16.67 mM and 833.33 U/mLmin, respectively. To our knowledge, this study reports, for the first time, the purification of PPO from *O. ficus-indica* by affinity column chromatography and the determination of *Km* and *Vmax* kinetic values in the world. As a result, the presence of PPO in the OFI was proven and the PPO activity was high. The PPO can be considered useful for many applications, such as nutritional, environmental, biomedical, and pharmaceutical. The information in this report on *O. ficus-indica* PPO purification will make it possible to study this enzyme in more detail.

## Figures and Tables

**Table 1:** Purification table of *O. ficus-indica* PPO.

Purification Steps	Volume (mL)	Activity (U/mLmin)	Total Activity	Total Protein (mg)	Specific Activity (U/mg Protein)	Purification Fold
Crude Extract	130	45.00	5850.00	3.7126	1575.72	
Ammonium sulfate precipitation	5	793.75	3968.75	1.1007	3605.68	2.29
Dialysis	7	217.50	1522.50	0.1748	8710.65	5.53
Affinity chromatography	2	105.00	210.00	0.0065	32,449.13	20.59

**Table 2:** Optimum temperature and pH values for the substrates.

Temperature (°C)	Catechol		4-Methyl Catechol		Pyrogallol	
	Optimum pH	Activity (U/mLmin)	Optimum pH	Activity (U/mLmin)	Optimum pH	Activity (U/mLmin)
15	7.5	108.75	6.0	150.00	4.5	293.75
20	7.5	173.33	5.5	140.75	9.0	2466.25
25	6.5	132.50	7.0	50.00	7.0	1640.00
30	5.0	83.33	7.5	330.00	6.5	131.25
35	9.0	35.00	6.5	41.68	8.5	442.50
40	8.5	125.00	7.5	97.50	6.0	172.50
45	5.0	162.50	6.5	85.00	6.5	201.25

**Table 3:** *Km* and *Vmax* values for the substrates.

Substrates	*Km* (mM)	*Vmax* (U/mLmin)	*Vmax*/*Km* (U/mLminmM)
Catechol	6.33	126.58	20.00
4-methyl catechol	5.38	107.53	20.00
Pyrogallol	16.67	833.33	50.00

## Data Availability

The data obtained in this study are available upon request from the corresponding author.
